# First Report of a Complete Genome Sequence of a Variant African Swine Fever Virus in the Mekong Delta, Vietnam

**DOI:** 10.3390/pathogens11070797

**Published:** 2022-07-15

**Authors:** Nguyen Duc Hien, Lam Thanh Nguyen, Le Trung Hoang, Nguyen Ngoc Bich, To My Quyen, Norikazu Isoda, Yoshihiro Sakoda

**Affiliations:** 1 Department of Veterinary Medicine, College of Agriculture, Can Tho University, Campus II, 3/2 Street, Ninh Kieu District, Can Tho 900000, Vietnam; ntlam@ctu.edu.vn (L.T.N.); lthoang1975@yahoo.com.vn (L.T.H.); bichm0320009@gstudent.ctu.edu.vn (N.N.B.); quyento777@gmail.com (T.M.Q.); 2 Can Tho Sub-Department of Animal Health, Ministry of Agriculture and Rural Development, 30/4 Street, Ninh Kieu District, Can Tho 900000, Vietnam; 3 Laboratory of Microbiology, Department of Disease Control, Faculty of Veterinary Medicine, Hokkaido University, North 18, West 9, Kita-ku, Sapporo 060-0818, Hokkaido, Japan; nisoda@vetmed.hokudai.ac.jp

**Keywords:** African swine fever, complete genome, Mekong Delta, variant, Vietnam

## Abstract

The objective of this study is to report the complete-genome sequence of a field African swine fever (ASF) virus (ASFV), namely ASF/VN/CanTho-OM/2021, which caused a fatal outbreak in domestic pigs in the Mekong Delta. Complete-genome sequencing detected an 18 bp nucleotide deletion in the EP402R gene (encoding for serotype-specific proteins CD2v) of ASF/VN/CanTho-OM/2021, which was determined to belong to genotype 2 and serotype 8. This mutation pattern was confirmed as unique in GenBank; thus, ASF/VN/CanTho-OM/2021 can be considered a novel variant, with a potential change of sero-characteristics within genotype 2. An additional unique mutation of 78 bp nucleotide insertion was also observed in the B475L gene. Additionally, four copies of tandem repeat sequences were found in the intergenic region (IGR) located between I73R and I329L, previously assigned as the IGR III variant. This study is the first to report the complete genome of ASFV in the Mekong Delta, and it highlights the necessity of strengthening molecular surveillance to provide further knowledge on the evolution and incursion of ASFV in the Mekong Delta and Vietnam.

## 1. Introduction

African swine fever (ASF) is a highly infectious disease in pigs caused by the African swine fever virus (ASFV), which might result in a high mortality rate approaching 100% [1,2]. ASFV is the sole member of the family *Asfarviridae*, a DNA arbovirus. The viral genome of ASFV has 170 to 195 kbp, encoding at least 160 open reading frames [3,4]. Currently, 24 genotypes and 8 serotypes have been reported worldwide based on the B646L gene encoding the major capsid protein p72 and the EP402R gene encoding the viral hemagglutinin CD2-like protein (CD2v) [5].

ASF was first reported in Kenya in 1921 [6]. Since the 1950s, ASFV has spread throughout Europe in two separate epidemic waves. The first wave in 1957 was through Spain and Portugal to other countries in Western Europe, and it was eradicated in many countries by the mid-1990s [1,7]. The second wave of the disease started in 2007, when ASF was detected in Georgia, and subsequently in neighboring countries in Eastern Europe; since then, ASF has become endemic in Russia and several European countries [8]. Since its first introduction to China in August 2018, ASF has spread rapidly to several other Asian countries [9]. Specifically, in Vietnam, the disease was first reported in February 2019 in the Northern provinces and became endemic afterward, causing serial outbreaks in domestic pigs across the country. Several previous studies have been conducted to determine the genotypes and serotypes of ASFV circulating in Vietnam and have confirmed that all detected ASFVs belong to genotype 2 and serotype 8 [10,11,12]. Currently, at least three variants of genotype 2 based on the variation of tandem repeat sequences (TRS) in the intergenic region (IGR) located between I73R and I329L genes have been identified in North Vietnam [13,14]. Moreover, other mutations might exist in the genome of circulating ASFVs, which might alter the virulence and sero-characteristics of field viruses; nevertheless, information on the complete genome of field ASFVs in Vietnam remains limited. Therefore, in this study, a causative ASFV of an outbreak in domestic pigs in the Mekong Delta in 2021 was subjected to next-generation sequencing (NGS), and this study revealed a novel variant based on EP402R (encoding for serotype-specific proteins CD2v) and B475L of the field ASFV.

## 2. Results

**Figure 1:**
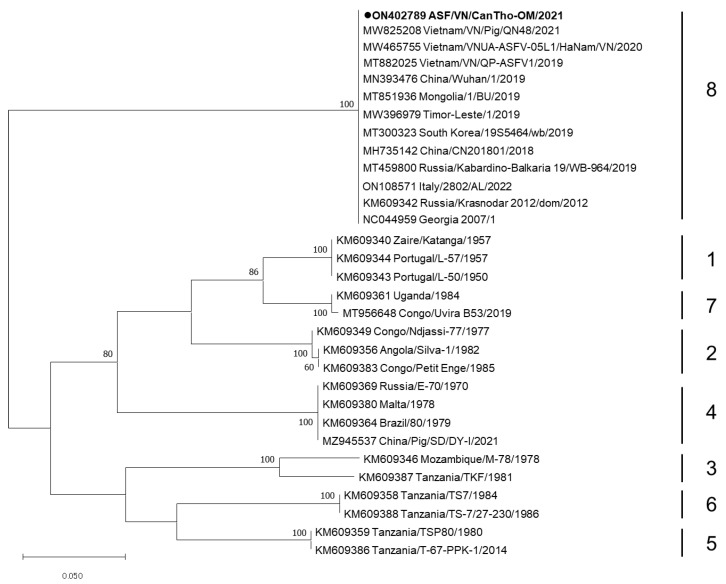
A maximum-likelihood phylogenetic tree based on the complete sequences of the EP402R gene (encoding for serotype-specific proteins CD2v) of ASFV. The Kimura 2-parameter model was used to construct the phylogenetic tree using MEGA 7.0. The numbers along the branches indicate bootstrap values of >70% (1000 replicates). The bars and numbers on the right indicate the ASFV serotypes. Black circles indicate the ASFV detected in this study, ASF/VN/CanTho-OM/2021, which caused an outbreak in the Mekong Delta in 2021.

**Figure 2:**
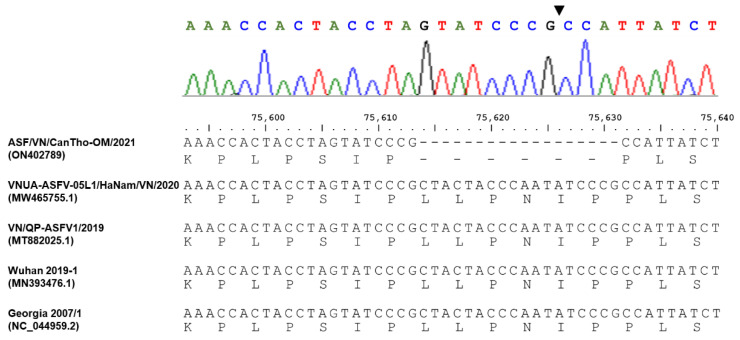
Alignment of the partial sequences in the EP402R of ASF/VN/CanTho-OM/2021 and other reference ASFVs showing an 18 bp nucleotide deletion of ASF/VN/CanTho-OM/2021. The top panel shows the chromatogram trace from Sanger sequencing for the partial nucleotide sequence containing the 18 bp nucleotide deletion in the EP402R gene of ASF/VN/CanTho-OM/2021. The black triangle indicates the deletion position.

The whole-genome sequence (WGS) of ASFV detected in this study, namely ASF/VN/CanTho-OM/2021, was deposited to GenBank and assigned the accession number ON402789. Based on the sequence alignment of the B646L gene (p72), ASF/VN/CanTho-OM/2021 showed 100% nucleotide identity with ASFVs previously detected in Vietnam, China and Georgia 2007/1, which all belong to genotype 2 (data not shown). Similarly, a phylogenetic analysis of the EP402R gene (CD2v) confirmed that ASF/VN/CanTho-OM/2021 belongs to serotype 8 (Figure 1). Interestingly, ASF/VN/CanTho-OM/2021 had an 18 bp nucleotide deletion of “CTACTACCCAATATCCCG” at positions 75,614 to 75,631 (based on the numbering scheme of NC_044959.2, Georgia 2007/1, Figure 2). The new sequence pattern of EP402R was confirmed as unique because there was no homology to other sequences previously deposited in GenBank (data not shown). This 18 bp nucleotide deletion results in a six-amino-acid deletion in the CD2v of ASF/VN/CanTho-OM/2021 (Figure 2).

**Figure 3:**
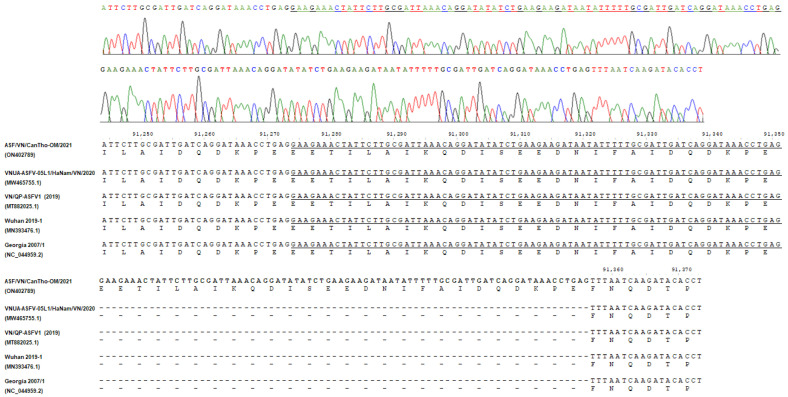
Alignment of the partial nucleotide sequences in the B475L of ASF/VN/CanTho-OM/2021 and other reference ASFVs showing a 78 bp insertion of ASF/VN/CanTho-OM/2021. The underlined and boldfaced characters indicate the original and repeated insertion sequences in the B475L of ASF/VN/CanTho-OM/2021, respectively. The top panel shows the chromatogram trace from Sanger sequencing for the partial nucleotide sequence containing the 78 bp insertion in the B475L gene of ASF/VN/CanTho-OM/2021.

Similarly, ASF/VN/CanTho-OM/2021 has a repeated insertion of 78-nucleotide sequence in the B475L gene “GAAGAAACTATTCTTGCGATTAAACAGGATATATCTGAAGAAGATAATATTTTTGCGATTGATCAGGATAAACCTGAG” at positions 91,352 to 91,356 corresponding to 26-amino-acid insertion (Figure 3). The repeated insertion was also first observed in GenBank, with no identical sequence previously deposited (data not shown).

**Figure 4:**
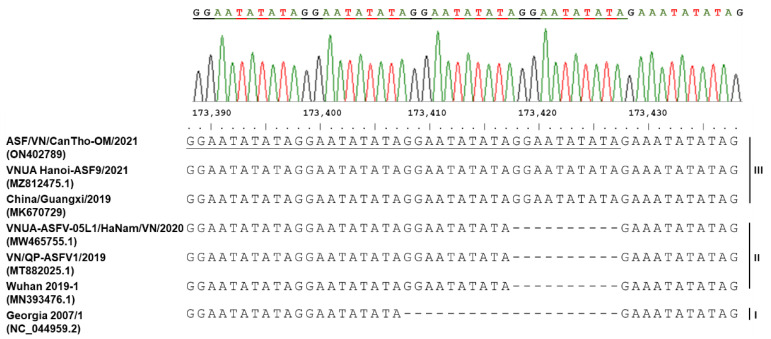
Alignment of the partial nucleotide sequences of the IGR between the l73R and I329L genes of ASF/VN/CanTho-OM/2021 and other reference ASFVs indicating four copies of TRS “GGAATATATA” (underlined characters) of ASF/VN/CanTho-OM/2021. The top panel shows the chromatogram trace from Sanger sequencing for the partial nucleotide sequence containing four copies of TRS of ASF/VN/CanTho-OM/2021.

In addition, the nucleotide sequence of the IGR between the I73R and I329L genes of ASF/VN/CanTho-OM/2021 contains four copies of TRS “GGAATATATA”, which are identical to the nucleotide sequence of VNUA/Hanoi-ASF9/2021 (MZ812475.1) and China/Guangxi/2019 (MK670729.1), which all belong to IGR III variant classification (Figure 4). Therefore, ASF/VN/CanTho-OM/2021 can be classified into the IGR III variant of genotype 2.

## 3. Discussion

In Vietnam, the first ASF outbreak was reported in February 2019. The disease quickly spread across all provinces, and ASF is currently endemic all over the country, with serial outbreak reports [10,13]. According to the Vietnam Department of Animal Health, from 1 January 2022 to 29 March 2022, a total of 562 ASF outbreaks were reported from 42 provinces in all northern, central and southern regions (https://www.fao.org/ag/againfo/programmes/en/empres/ASF/situation_update.html, accessed on 1 May 2022).

Owing to the importance and widespread distribution of ASF in Vietnam, many studies have been conducted for the genetic characterization of circulating ASFVs and tracing incursion of viruses based on specific molecular markers [10,11,12]. Most focused on ASFVs detected in the northern region of Vietnam, but limited information on ASFVs circulating in the Mekong Delta, the southernmost region comprising 1 central city and 12 provinces, is available. In this study, a causative ASFV for a fatal outbreak in domestic pigs in the Mekong Delta was subjected to complete-genome analysis. In October 2021, a backyard pig farm suspected with ASF was reported in Can Tho city, which is considered the epicenter of the Mekong Delta. The presence of ASFV was first confirmed using conventional PCR, which targeted the B646L gene. Subsequently, the complete genome of ASFV, ASF/VN/CanTho-OM/2021, was obtained using NGS to better understand the evolution and incursion of ASFV in the region.

The phylogenetic analysis based on B646L (p72) and EP402R (CD2v) sequences indicated that ASF/VN/CanTho-OM/2021 belongs to genotype 2 and serotype 8 (Figure 1). This result was consistent with previous reports showing that all ASFVs causing outbreaks in Vietnam belong to genotype 2 and serotype 8 [10,11,12]. This is the most predominant genotype/serotype of invasive ASFVs currently circulating in several Asian and European countries, all derived from the invasive Georgia/2007/1 isolate [15].

Intriguingly, an 18 bp nucleotide deletion of “CTACTACCCAATATCCCG” was detected in the EP402R gene, which resulted in a six-amino-acid deletion “LLPNIP” in the CD2v of ASF/VN/CanTho-OM/2021 (Figure 2). The short mutation sequence in the EP402R was confirmed as unique because it has no homology to other sequences previously deposited in GenBank (data not shown). Nevertheless, this mutation was not detected in the phylogenetic tree of EP402R because gaps resulting from a deletion in the DNA alignment were treated as missing data; therefore, it was not accounted for in statistical estimation in common phylogenetic analyses. A previous study also argued that phylogenetic analysis of several important genes of ASFV, such as B646L (p72) and EP402R (CD2v), did not accurately define the genotypes and serotypes of ASFVs [16]. Thus, this study remains limited as it did not fully elucidate how the deletion affects the sero-characteristics of the virus.

Based on complete-genome analysis, this study detected another unique mutation in the B475L that had a repeated insertion of the 78-nucleotide sequence in the B475L gene “GAAGAAACTATTCTTGCGATTAAACAGGATATATCTGAAGAAGATAATATTTTTGCGATTGATCAGGATAAACCTGAG” (Figure 3). In fact, the function of B475L remains unknown. Therefore, further studies and information are needed to determine the role of the gene and the causation of the mutation in the B475L to improve the understanding of the pathogenicity and evolution of ASFVs. On the other hand, it is believed that ASF/VN/CanTho-OM/2021 is highly virulent in domestic pigs because infected animals display obvious clinical symptoms of acute ASF infection, such as hyperthermia, respiratory distress, skin hemorrhages, hemorrhagic splenomegaly and lymph node and multiorgan hemorrhages.

The sequence of TRS insertions in IGR between the I73R and I329L genes is widely used as a genome marker to discriminate closely circulating ASFVs worldwide, including in Vietnam [14,17,18]. The comparative DNA alignment based on the IGR between the I73R and I329L genes showed that TRS insertions of ASF/VN/CanTho-OM/2021 are similar to other ASFVs detected in northern Vietnam and China (Figure 4). It is assumed that the widespread distribution of the IGR III variant is a result of the incursion of ASFVs in China to several other Asian countries, including Vietnam [14,17,18]. Currently, at least three different variants of ASFV (IGR I, II and III) circulate in the domestic pig population in Vietnam [13]. Detection of circulation of the IGR III variant might provide important information for the incursion of ASFV in the Mekong Delta, which is more likely from the northern to southern regions in Vietnam [12,13]. It is also noteworthy that no nucleotide mutation was found in the other major functional genes of ASF/VN/CanTho-OM/2021, such as O61R (p12, attachment protein), CP204L (p30, phosphoprotein involved in virus entry) and E183L (p54, binds to the LC8 chain of dynein involved in virus entry; Supplemental Figure S1).

This study is the first to report the complete genome of ASFV in the Mekong Delta and detect a distinct variant of genotype 2. The disease remains highly endemic in the region, but little information is known about circulating viruses. Therefore, this study provides great significance for understanding the epidemiology, evolution and incursion of ASFV in the Mekong Delta and Vietnam.

## 4. Materials and Methods

### 4.1. Sample Collection and Sampling Site

This study was conducted following a fatal virus outbreak in domestic pigs on a backyard farm in Can Tho city, a central administrative division of the Mekong Delta with a distance of about 1500 km from Ha Noi capital, in October 2021. All 11 infected pigs displayed obvious clinical symptoms of ASF infection. The samples were collected from organs (spleen and lymph nodes) of 2 dead pigs out of the 11 clinically infected pigs at the farm and used for conventional PCR targeting of the B646L gene to confirm the presence of ASFV, as described previously [5].

### 4.2. Whole-Genome Sequencing

NGS was used to obtain the WGS of the detected ASFV. Briefly, total viral DNAs were directly extracted from the collected organs using the Wizard^®^ Genomic DNA Purification Kit (Promega, Madison, WI, USA). The WGS was performed at the KTest Science Company (Ho Chi Minh City, Vietnam) using the Illumina MiniSeq platform (2 × 150 bp paired ends). Adapters, primers and low-quality sequences (average score < 20 and read length < 35 bp) were removed using fastp version 0.20.1 [19]. The trimmed reads were mapped to the reference genome Sscrofa11 from Ensembl (GCA_000003025.6) using bowtie2 version 2.4.2 [20] with default settings. The unmapped reads were de novo assembled using Unicycler version 0.4.8 [21] with default settings to generate ASFV genomes. Geneious Prime 2021.2.2 was used for scaffolding and annotation of the assembled ASFV genome using Georgia 2007/1 (GenBank accession number NC_044959.2) and Wuhan/2019 (GenBank accession number MN393476) as reference genomes.

### 4.3. DNA Alignment and Phylogenetic Analysis

The complete genome in a single contig was submitted for downstream analyses such as DNA multiple alignments, trimming and phylogenetic tree construction. The full-length genome sequence of ASFV detected in this study and other ASFV reference strains obtained from GenBank were aligned using MAFFT version 7 with default settings [22]. The quality of the alignment was manually inspected using BioEdit version 7.0.544. A maximum-likelihood tree for each B646L and EP402R sequence was constructed using MEGA 7.0 with a resampling process of 1000 replicates. The manipulation of the DNA sequence was carried out using R version 3.4.4 with the contributed packages *ape* [23], *ips* [24] and *msa* [25].

### 4.4. PCR Amplification and Sanger Sequencing

Sequences in the genome contig of ASF/VN/CanTho-OM/2021 containing a mutation comparable to the Georgia 2007/1 genome were amplified by conventional PCR and sequenced using the Sanger method for verification. DNA alignments and chromatogram traces from Sanger sequencing were manually inspected using BioEdit version 7.0.5 [26].

## Data Availability

The complete-genome sequence generated in this study was submitted to GenBank under accession number: ON402789. Data that support the findings of this study are available upon request from the authors.

## Supplementary Materials

The following supporting information can be downloaded at: https://www.mdpi.com/article/10.3390/pathogens11070797/s1, Figure S1: Alignments of the full nucleotide sequences of (A) O61R gene (p12, attachment protein); (B) CP204L gene (p30, phosphoprotein involved in virus entry) and (C) E183L gene (p54, binds to the LC8 chain of dynein involved in virus entry) of ASF/VN/CanTho-OM/2021 and other reference ASFVs.
