# Supplementary material for: First Report of a Complete Genome Sequence of a Variant African Swine Fever Virus in the Mekong Delta, Vietnam
Source: Pathogens. 2022 Jul 15;11(7):797. doi: 10.3390/pathogens11070797 (PMC9318516; doi:10.3390/pathogens11070797)

Communication

First report of a complete genome sequence of a variant African swine fever virus in the Mekong Delta, Vietnam

Nguyen Duc Hien[1,2]*, Lam Thanh Nguyen[1], Le Trung Hoang[1,2], Nguyen Ngoc Bich[1], To My Quyen[1], Norikazu Isoda[3] and Yoshihiro Sakoda[3]*

[1] Department of Veterinary Medicine, College of Agriculture, Can Tho University, Campus II, 3/2 street, Ninh Kieu district, Can Tho 90-0000, Vietnam; ndhien@ctu.edu.vn (N.D.H.); ntlam@ctu.edu.vn (L.T.N.); lthoang1975@yahoo.com.vn (L.T.H.); bichm032009@gstudent.ctu.edu.vn (N.N.B.); quyento777@gmail.com (T.M.Q.)

[2] Can Tho Sub-Department of Animal Health, Ministry of Agriculture and Rural Development, 30/4 street, Ninh Kieu district, Can Tho 90-0000, Vietnam

[3] Laboratory of Microbiology, Department of Disease Control, Graduate School of Veterinary Medicine, Hokkaido University, North 18, West 9, Kita-ku, Sapporo 060-0818, Hokkaido, Japan; nisoda@vetmed.hokudai.ac.jp (N.I.); sakoda@vetmed.hokudai.ac.jp (Y.S.)

* Correspondence: ndhien@ctu.edu.vn (N.D.H.); Tel.: +84-292-384-0371; Fax: +84-292-384-037; Department of Veterinary Medicine, College of Agriculture, Can Tho University, Campus II, 3/2 street, Ninh Kieu district, Can Tho 90-0000, Vietnam

* Correspondence: sakoda@vetmed.hokudai.ac.jp (Y.S.); Tel.: +81-011-706-5207; Fax: +81-1-1706-5273; Laboratory of Microbiology, Department of Disease Control, Graduate School of Veterinary Medicine, Hokkaido University, North 18, West 9, Kita-ku, Sapporo 060-0818, Hokkaido, Japan

Supplementary figure legend

Supplementary Figure S1: Alignments of the full nucleotide sequences of A) O61R gene (p12, attachment protein); B) CP204L gene (p30, phosphoprotein involved in virus entry) and C) E183L gene (p54, binds to the LC8 chain of dynein involved in virus entry) of ASF/VN/CanTho-OM/2021 and other reference ASFVs. "•" indicates the same nucleotides as ASF/VN/CanTho-OM/2021.

Supplementary Figure S1-A: O61R gene (p12, attachment protein)

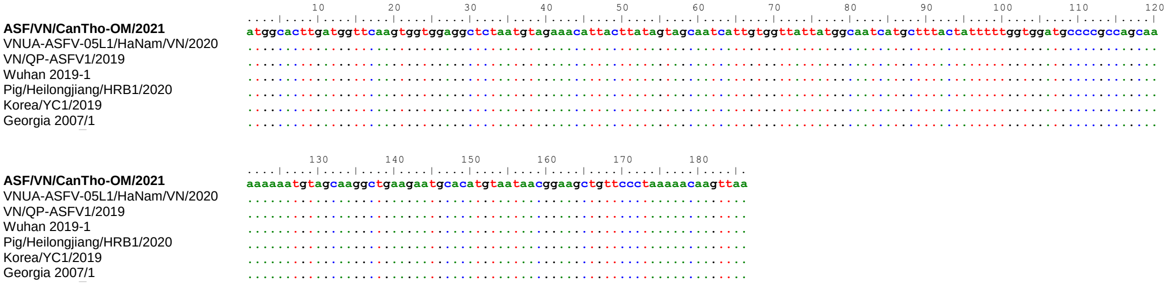

Supplementary Figure S1-B: CP204L gene (p30, phosphoprotein involved in virus entry)

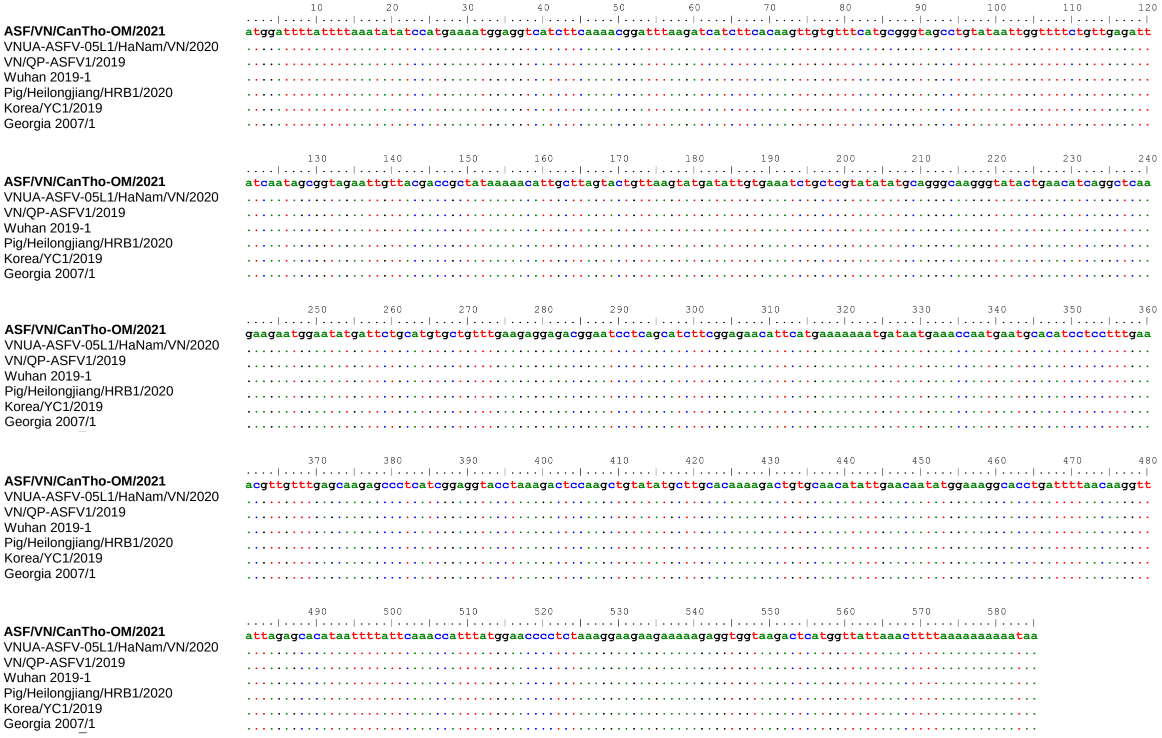

Supplementary Figure S1-C: E183L gene (p54, binds to the LC8 chain of dynein involved in virus entry)

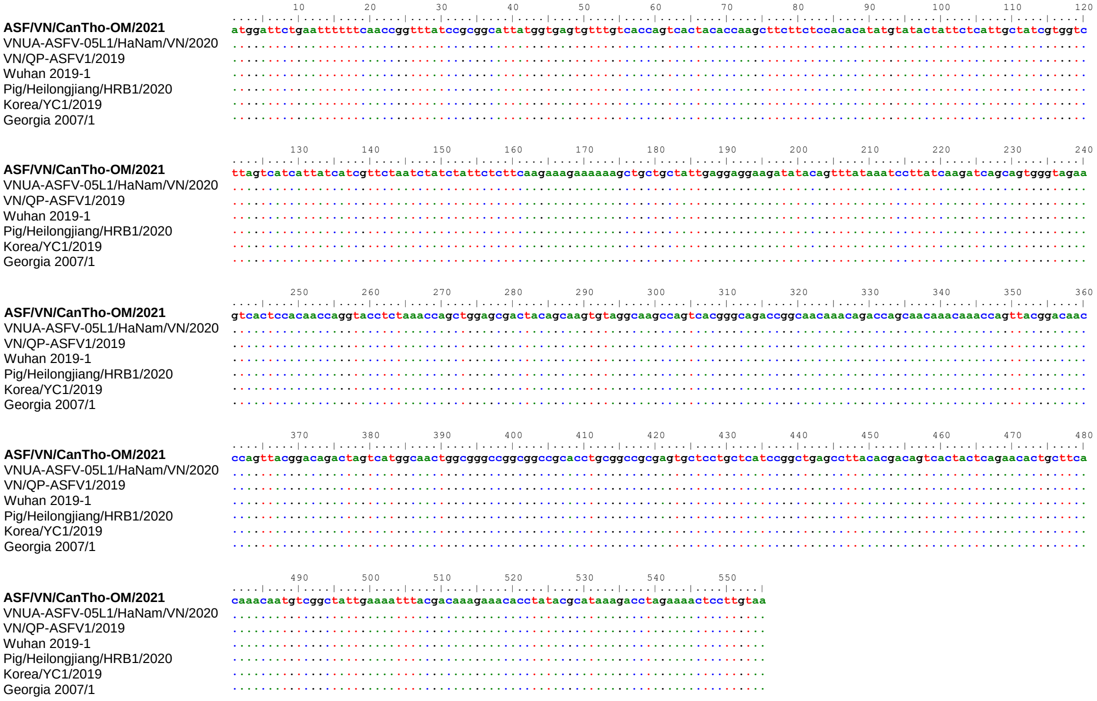

Supplement: Supplementary file 1 [file pathogens-11-00797-s001.zip › pathogens-1793478-supplementary.pdf]
